# As One and Many: Relating Individual and Emergent Group-Level Generative Models in Active Inference

**DOI:** 10.3390/e27020143

**Published:** 2025-02-01

**Authors:** Peter Thestrup Waade, Christoffer Lundbak Olesen, Jonathan Ehrenreich Laursen, Samuel William Nehrer, Conor Heins, Karl Friston, Christoph Mathys

**Affiliations:** 1 Interacting Minds Centre, Aarhus University, 8000 Aarhus, Denmark; ptw@cas.au.dk (P.T.W.); clo@cas.au.dk (C.L.O.); chmathys@cas.au.dk (C.M.); 2 School of Communication and Culture, Aarhus University, 8000 Aarhus, Denmark; 202204836@post.au.dk (J.E.L.); 202204724@post.au.dk (S.W.N.); 3 Department of Collective Behavior, Max Planck Institute for Animal Behavior, 78457 Konstanz, Germany; 4 Queen Square, Institute of Neurology, University College London, London WC1N 3AR, UK; k.friston@ucl.ac.uk

**Keywords:** active inference, free energy principle, Markov blanket, predictive processing, cognitive modelling, multi-scale, collective intelligence, emergence, 87.15.Aa, 91-08, C63

## Abstract

Active inference under the Free Energy Principle has been proposed as an across-scales compatible framework for understanding and modelling behaviour and self-maintenance. Crucially, a collective of active inference agents can, if they maintain a group-level Markov blanket, constitute a larger group-level active inference agent with a generative model of its own. This potential for computational scale-free structures speaks to the application of active inference to self-organizing systems across spatiotemporal scales, from cells to human collectives. Due to the difficulty of reconstructing the generative model that explains the behaviour of emergent group-level agents, there has been little research on this kind of multi-scale active inference. Here, we propose a data-driven methodology for characterising the relation between the generative model of a group-level agent and the dynamics of its constituent individual agents. We apply methods from computational cognitive modelling and computational psychiatry, applicable for active inference as well as other types of modelling approaches. Using a simple Multi-Armed Bandit task as an example, we employ the new ActiveInference.jl library for Julia to simulate a collective of agents who are equipped with a Markov blanket. We use sampling-based parameter estimation to make inferences about the generative model of the group-level agent, and we show that there is a non-trivial relationship between the generative models of individual agents and the group-level agent they constitute, even in this simple setting. Finally, we point to a number of ways in which this methodology might be applied to better understand the relations between nested active inference agents across scales.

## 1. Introduction

Active inference [1,2,3], under the Free Energy Principle [4,5,6,7,8] has been proposed as a formal framework generally applicable for modelling living and adaptive systems across spatial and temporal scales of description [9]. From this perspective, perception, learning, action, and cognition in general can be described as approximate Bayesian inference driven by a single optimization process, contingent on a generative model of the environment. Crucially, a system can be considered to perform active inference once it maintains a causal boundary—a Markov blanket. It has been suggested that collectives of agents individually performing active inference can maintain such a boundary, leading to a hierarchy of nested simultaneous processes of active inference [10,11]. This potential for nested applicability underwrites much of active inference’s ubiquity, and several simulation studies have investigated the emergent processes which ensue. Despite this, very few studies have been able analytically to relate the generative model of a group-level active inference process to the generative models of the individual agents [12]. Here, we propose a method for doing this, based on a kind of cognitive modelling inspired by computational psychiatry. In the following, we briefly introduce active inference and the Free Energy Principle, focusing on how it has been applied to multi-agent and multi-scale contexts. We then demonstrate our approach in a simple case, showing how emergent group-level active inference processes have non-trivial relations to their constituent parts. Finally, we discuss the applicability of this approach more broadly.

### 1.1. Active Inference and the Free Energy Principle

The Free Energy Principle [4,5,6,7,8] is the claim that any system that maintains a stable boundary—formalized as a Markov blanket—can be described as minimizing the variational free energy of its sensory states. A Markov blanket is a set of states that renders states internal to it conditionally independent of external states. Blanket states are further divided into sensory states, which affect—but are not affected by—the internal states and active states, which affect—but are not affected by—external states. The ensuing separation of internal and external states by blanket states (i.e., conditional independence) is statistical, and not necessarily also causal [13]. In state-space formulations, the Free Energy Principle has additionally rested on the assumption that the system is at a non-equilibrium steady state, which is unnecessary in path-integral formulations [8,14]. Maintaining a Markov blanket can be shown to imply a minimization of a negative log-probability (i.e., potential energy or self-information)—called the surprise *ℑ*—of the sensory states, given a generative model, that is, one of how states in the environment generate those states. Surprise is not computable in most cases of interest but can be approximated by a variational free energy upper bound; minimization of surprise then becomes feasible by minimizing the variational free energy. Minimizing variational free energy is equivalent to performing variational (approximate) Bayesian inference, which enables a description of systems that maintain Markov blankets as engaging in active inference (viz., self evidencing).

Active inference is the Free Energy Principle applied to perception, learning, and action. Here, perception is viewed as variational inference on the state of the environment, given sensory states and a generative model, which can itself be updated by Bayesian parameter learning and Bayesian model selection (viz., structure learning). Here, inference corresponds to optimizing a posterior distribution (with an assumed functional form) that has the lowest associated variational free energy given the sensory inputs, which can be shown to be the variational posterior that best approximates the true Bayesian posterior given the constraints imposed on its functional form (e.g., Gaussianity). Actions (or often policies—sequences of actions with some specified temporal depth) are then selected that minimize the expected free energy, namely, the expectation of the free energy under predicted sensory outcomes, given those actions. This affords a view of action selection as a type of inference. Perception and action thus constitute the two possible ways of minimizing free energy: changing one’s expectations and changing the world (and thereby the sensory observations). Active inference models come equipped with a preference prior—a prior expectation for the types of sensory states the agent is likely to encounter. The preference prior is usually immutable, which means that the only way to minimize free energy is to act such that the prior is most likely to be realized, and that it therefore encodes the preferred observations of the agent. Technically, these preferred states in general constitute the attracting states of the agent’s dynamics, namely, the kind of states that are characteristic of the agent at hand.

Active inference can be applied as a behavioural model irrespective of the Free Energy Principle. It has been applied in a variety of fields, either to understand or to build adaptive systems, including theoretical neurobiology and neuroscience [15], cognitive science and computational psychiatry [16,17], robotics and machine learning [18], and philosophy of mind [19,20]. It generalizes a variety of related approaches like reinforcement learning, KL-control, and expected utility maximization [1,21]. Whether active inference is used to model observed systems or build artificial ones, in order to apply it in any specific context, the first task is to specify a generative model, which provides the constraints that a resulting (active) inference process obeys (i.e., specifies the attracting set of states that characterize the agent). Many types of generative models are used in active inference, including continuous as well as discrete state-space models and models made for specific contexts as well as more generally applicable models. Here, we specifically employ a widely used discrete state-space model, the Partially Observable Markov Decision Process (POMDP) (see Section 2.1 for a full description).

Active inference under the Free Energy Principle is sometimes presented as taking place in nested Markov blanket structures, where smaller agents compose larger-scale blankets that become agents in their own right [10,22]. These group-agents can then in turn be part of even larger-scale blanket structures—like cells forming organs, which in turn form human bodies and eventually human collectives. In this view, an obvious question to ask is how the active inference agents at higher levels relate to the activity and interactions of the smaller-scale agents that constitute them. We here demonstrate a method for investigating exactly this, based on cognitive modelling. In the following section, we preface the introduction of this method with an overview of the literature to date of active inference in multi-agents settings.

### 1.2. Multi-Agent and Collective Active Inference

Since its inception, Active Inference has been applied to “social” contexts, that is, contexts where multiple agents interact (e.g., [23,24]). Work on multi-agent active inference traditionally unfolds on one or more of three scales of description: a *within-agent scale*, where the focus is to investigate what kinds of generative models are appropriate for interacting with environments containing other agents; a *between-agent* scale, where the focus is to understand how interactions between multiple agents—ranging in number from dyads to whole populations—mutually shape their behavioural and belief dynamics over time; and a *group-as-agent* scale, where a collective of agents forms an emergent group that possesses a Markov blanket of its own, and which therefore instantiates an active inference agent in its own right. Work has been conducted on all three scales, but we are not aware of any work that manages to reconstruct the generative model of an emergent group-level agent, or to compare it with the dynamics of its constituent agents at the two lower levels. In the following, we give a brief overview of the literature on multi-agent active inference across the three levels and proceed to describe how the work presented here complements previous work with a method for accessing the generative models of group-level agents.

A number of theoretical points have been made regarding the types of generative models that must be held by social agents to function. It has been pointed out, for example, that the main statistical regularities in the environment—the parts that are most important to represent properly in a generative model—are social, specifically regarding other agents’ expectations for one’s own behaviour [25,26]. These expectations, which determine the most appropriate way to act in a given situation, can be inferred by attending to socially constructed cues (or “cultural affordances”) in the environment [27]—cues that provide (and are created to provide) what has been called “deontic value” in being informative about obligatory social rules [28].

It is possible to make inferences about the actions of others by selecting among explicit models of the mental processes underlying their behaviour [29] and allow it to affect one’s behaviour in collaborative tasks [30]—or even to use recursive Theory of Mind models where the level of recursion has to be explicitly limited [31,32]. This allows for explicit perspective-taking and interacting with agents different from oneself, but it is a complex and computationally costly process that may often not be necessary for coordination. It is argued that humans have evolved prior preferences for interacting with others whom they are mentally aligned with, that is, with whom they have similar expectations about the shared environment [33]—“shared protentions” in Husserlian terminology [34]—which facilitates communication and coordination in its own right. This is built on canonical simulation work showing that agents can, if they have similar generative models (also called a “shared narrative” [35]), make inferences about each others’ mental states without needing to explicitly model the (infinitely recursive) mutual perspective taking of the interaction [24] and that these linked active inference agents reach a free energy minimum over time by aligning their generative models [35]. The resulting generalized synchrony—if it is successfully instantiated, which is not always the case [36]—allows agents to communicate their beliefs about the environment to each other [37] and acquire a shared language that allows them to combine knowledge from complementary perspectives (a type of distributed intelligence called “federated inference”) to, for example, track a moving target (in order to hide from it) [38]. This type of generalized synchrony of strategies and belief states can also underpin the coordination of goal-oriented joint action for dyads [39] and teams of agents [40] or be a mechanism determining behaviour in competitive games like the prisoner’s dilemma [41]. On a multi-agent population level, the emergence of generalized synchrony—and the preference for being aligned with interlocutors—is observed in in vivo neurons [42,43,44] and can be a mechanism for implementing cumulative culture [45,46], which can also lead to separate echo-chamber-like epistemic communities that maintain highly precise and difficult-to-change beliefs [47].

The work mentioned above provides important clues for how successful social interaction can be underpinned by active inference—for biological as well as artificial and mixed intelligence systems [48]. It does not, however, engage directly with the proposed multi-scale nature of active inference, because it remains at a within- and between-agent level of description. There is also a strand of work regarding how a collective of (potentially active inference) agents can form an emergent whole with a Markov blanket of its own—which then, of course, can be considered an active inference agent in its own right. The canonical work here is a “primordial soup” simulation, where it is shown that a system maintaining a Markov blanket leads to the system’s internal states carrying information about external states as they come to minimize a variational free energy functional of the blanket states, and therefore model the environment [23]. One way to establish this type of boundary (as in the case of organic morphogenesis) is for the members of the collective to be equipped with a prior expectation of being part of such a structure [49], with cells creating and maintaining the larger structure (as well as differentiating into different roles, or cell types) in the process of reducing free energy [50]. These types of maintained Markov blanket structures can be nested within each other [22] in ways that are found in the brain [51] and which can be related to mental phenomena like the psychopathology of the emergent human mind [52]. There is also work on emergent Markov blankets not related to the brain, which does not rely on having explicit prior expectations for being in a specific larger structure. Joint free energy minimization can be a mechanism for ant-like agents collectively solving a T-maze [53] or for the self-organization of collective motion whereby single particles can collectively synchronize their movements by only maintaining a goal prior over simple metrics like relative distance to neighbours [54]. Dyads of agents moving to target locations are designed to form collectives that could be considered to perform Bayesian inference relative to certain sensory states [55], and certain parameter regimes of spin-glass systems can be analytically related to a collective of active inference agents, whose joint actions implement Bayesian inference at a higher level (namely, via a form of sampling-based inference) [12].

The above work engages with how collectives of agents can come to form larger-scale Markov blanket structures, how they come to solve tasks as a group, and how to relate the dynamics of the agents—forming the internal states of the collective—to Bayesian inference happening at the collective level, all implying that there is an active inference process instantiated at the collective or group level. Despite this, previous studies have never explicitly modelled the inversion of the emergent group’s generative model and corresponding action selection and therefore have never considered generative models at multiple levels simultaneously. That is, they largely address inference at within- and between-agent levels, only hinting at group-level active inference proper. The main exception here is the spin glass simulation work by Heins and colleagues [12], where a group-level generative model is analytically related to the activity of constituent agents, although in a way limited to a relatively specific context, and without direct relation to an environment. Fully engaging with a multi-scale active inference account—and the particularly interesting question how the dynamics of the constituent agents relate to the group-level active inference process (for example in the case of psychopathology [52])—should entail relating the generative models at the different levels to each other (since they define the active inference process), and to the environment.

There are various reasons why this has not been addressed. Firstly, there is not always a well-defined environment to act on and be influenced by per se (as in Refs. [49,55]) or there might not be easily differentiated internal and blanket states (as in Refs. [38,53,54]) to implement the inference. A more important reason, however, is probably the fact that the generative model of the group-level agent is difficult to access. As opposed to the generative models of the constituent agents, which are specified by the modellers and therefore known, the generative model of the group is emergent and a priori unknown. This means it has to be reconstructed in order to be compared to the constituent agents, which is not a trivial task. There exist, however, some standard methods for attempting to reconstruct unknown generative models of systems. One such approach comes from cognitive modelling, a field where the primary task is to infer on the unknown generative models underlying observable behaviour, often of human subjects. From the perspective of active inference, observable behaviour is simply the blanket states of the system. This fits well with cognitive and behavioural modelling, where a Markov blanket-like structure is usually assumed. Here, we use this approach to make inferences about the generative model of a group-level agent. This group-level agent comprises a set of agents in a predefined Markov blanket structure and with a simple internal structure and external environment. This allows us to use the same generative model structure for the individual agents and the group-level agent. After confirming that the proposed method works for this specific simulation, we go on to make preliminary investigations of the relations between group- and individual-level generative models. The approach we present here should be applicable in many contexts including when boundaries are dynamically established or when internal structures and external environments are more complex.

## 2. Materials and Methods

Here, we introduce a methodology for relating generative models and ensuing active inference across levels of nested Markov blankets based on fitting active inference models to the behaviour of the group-level agent in the fashion of computational cognitive modelling. The method is applicable as long as (i) there are clearly definable blanket states (observations and actions) for the group-level agent at each time point; (ii) one or more appropriate generative model structures for the environment of the group-level agent can be identified; and (iii) the resulting behaviour (i.e., blanket states) of the group-level agent is informative, that is, different types of generative models (or parameters of generative models) can be distinguished based on the behaviour. In our illustrative case, we ensure the first condition by composing agents in a fixed Markov blanket structure, and the second and third by choosing a group-level environment for which there are existing well-defined generative models: namely, the Multi-Armed Bandit task (MAB). In addition, this allows us to use the same generative model for the internal agents of the group as for the group level, which means that we can validate the method by confirming a priori expected relations. That being said, the method is also applicable in principle with emergent Markov blankets and with more complex environments.

In the following, we describe the Multi-Armed Bandit task active inference model we use (Section 2.1), the fundamentals of computational cognitive modelling (Section 2.2), and the construction of the group-level agent (Section 2.3). Finally, we report the numerical experiments we used as preliminary investigations into the relations between the two levels of description (Section 2.4). All simulations were performed in Julia (version 1.10) [56], relying primarily on a new library for simulating and fitting active inference models, ActiveInference.jl (version 0.0.2) [57] as well as its sister library for fitting behavioural models to data, ActionModels.jl (version 0.5.0), itself an extension to the powerful Turing.jl library (version 0.10) [58] for Bayesian parameter estimation. All code and synthesized data are publicly available at https://osf.io/6z3bd/.

### 2.1. Active Inference for Multi-Armed Bandit Tasks

The Multi-Armed Bandit (MAB) task is a ubiquitous task in cognitive science and neuroscience as well as in machine learning [59]. It is simple to design and model but can stand in for a wide range of environments that depend probabilistically on an agent’s actions. As one of the simplest possible instantiations of a complete action-perception loop, it is suitable for establishing the method used here. A MAB task presents an agent (human or artificial) with an environment comprising a “bandit” with multiple arms (in our case three) which the agent can, in a trial-by-trial fashion, select among. Choosing a bandit arm probabilistically results in an outcome: here, we use binary outcomes, but they can in principle also be continuous or categorical. Traditionally, outcomes are referred to as “wins” and “losses”, and the agent’s task is to maximize the wins and minimize the losses it receives. In active inference, the value of a specific outcome is defined by a subjective preference which is specific to the agent: we therefore simply call them outcomes 1 and 2.

**Figure 1:**
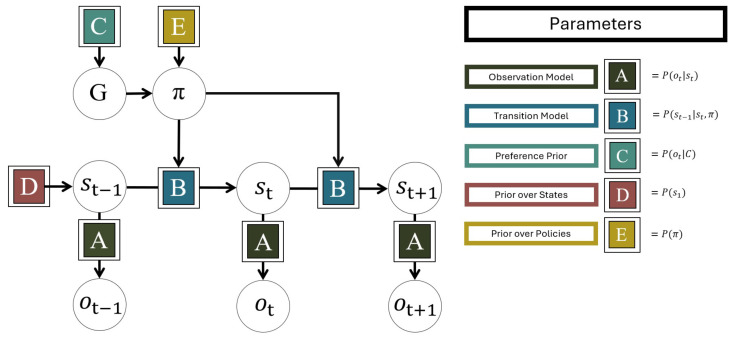
The generative model used for active inference based on a Partially Observable Markov Decision Process. Observations *o* depend on hidden states *s* according to the observation model *A*. *s* changes over time according to the transition model *B* and the chosen policy π. *C* is the preference prior which determines the expected free energy *G*. *D* and *E* provide priors for the hidden states and the policies, respectively.

Active inference has already been applied to MAB tasks [60], albeit with a specialized generative model. We here instead use a classic model type that is widely used in the active inference literature: the Partially Observable Markov Decision Process (POMDP) [1] (Figure 1). In the following, we give a brief technical overview of active inference with POMDP’s. For a comprehensive description of formal and implementational details of the POMDP-based active inference models used here, we refer to [57] as well as to more general technical introductions [1,61].

The POMDP is a flexible type of generative model for discrete state spaces in discrete time. POMDP-based generative models for active inference assume that at a given time *t*, a discrete observation $o_{t}$ depends on concurrent discrete hidden environmental states $s_{t}$, which evolve over time, conditioned on a policy π—a sequence of discrete actions $a_{t}$—as parametrized by five matrices *A-E*. The dependence of $o_{t}$ on $s_{t}$ is governed by the *A* matrix, sometimes called the observation model, which specifies the likelihood of making observations $o_{t}$ given each possible state of the environment $s_{t}$. The dynamics in $s_{t}$ is governed by the *B* matrix, sometimes called the transition model, which is a transition probability matrix over the possible states of $s_{t}$, given previous hidden states $s_{t-1}$. The preference prior is encoded by the *C* matrix, also called the *prior preferences* or the *goal prior*, which encodes a fixed a priori expectation over sensory outcomes and thereby define the agent’s preferences. The *D* matrix defines the agent’s prior over environmental states $s_{t}$, and the *E* matrix defines the agent’s prior over policies π, in effect defining habits that the agent returns to in the absence of other action incentives. The actions $a_{t}$ of the policy π select between different possible *B* matrices, thereby controlling the evolution of environmental states $s_{t}$.

Given a POMDP generative model p(o,s) of how environmental states change and give rise to sensory observations, perception can be implemented as variational Bayesian inference on the current environmental state. This is conducted by using gradient descent to choose an approximate posterior q(s) with the lowest variational free energy F (Equation (1)):

(1) $$F\left[q\left(s\right),o\right]≜D_{\mathrm{KL}}\left[q\left(s\right)∥p\left(o,s\right)\right]=\underset{\mathrm{Divergence}}{\underbrace{D_{\mathrm{KL}}\left[q\left(s\right)∥p\left(s|o\right)\right]}}\underset{\mathrm{Surprise}}{\underbrace{-\ln p(o)}}$$

Contrary to the true posterior p(s|o), F only depends on q(s) and p(o,s), so it can be calculated and minimized. When rewritten as the sum of the sensory surprise ℑ≜−lnp(o) and the divergence of q(s) from the true posterior, it can be seen how minimizing F by varying q(s) leads to the best approximation of the true posterior, resulting in the perceptual belief about the environmental state that caused the sensory observation.

Action selection and planning then consists of choosing the action policy π associated with the lowest expected free energy *G*, given the expected trajectory of states $\tilde{s}$ and observations $\tilde{o}$ (Equation (2)):

(2) $$G_{\pi }=-\underset{\mathrm{Information}\;\mathrm{gain}}{\underbrace{E_{q(\tilde{o},\tilde{s}|\pi )}\left[\ln q\left(\tilde{s}|\tilde{o},\pi \right)-\ln q\left(\tilde{s}|\pi \right)\right]}}-\underset{\mathrm{Pragmatic}\;\mathrm{value}}{\underbrace{E_{q(\tilde{o}|\pi )}\left[\ln p\left(\tilde{o}|C\right)\right]}}$$

The expected free energy *G* is calculated for a specific policy π as the negative sum of the expected change in beliefs (the information gain) and the expected conformity with the preference prior *C* (the pragmatic value). This brings about a natural explore–exploit balance, where actions are chosen that are expected to lead to observations which are informative about the environment but also similar to *C*, with the balance between the two being controlled by the precision of *C*. A softmax is then applied to the negative expected free energies for each policy $G_{\pi }$, after which it is multiplied with the prior over policies *E* to form the posterior over policies. The precision or inverse temperature of the softmax, γ, controls the weight of $G_{\pi }$ relative to *E*. Finally, the policy posterior is marginalized to only specify probabilities over the next actions, after which a softmax is applied to form the final action selection probabilities. We here specifically highlight the precision or inverse temperature of this softmax, the α parameter, since it is the target of the simulation studies presented in this article. Higher α values lead to more deterministic action selection, i.e., the agent always selects the most likely action, as opposed to probability matching (at α=1) or to random choices independent of beliefs (as α gets lower). Notably, when *E* is uniform (i.e., actions are selected at random in the absence of preferences for policies), γ and α have similar effects on behaviour. Many additional types of parametrizations can be used to extend the model (e.g., to construct hierarchically, counterfactually, and temporally deep models [62,63] or to enable parameter learning [1]), which we do not employ here. See [1] for a full account of the various possibilities.

**Figure 2:**
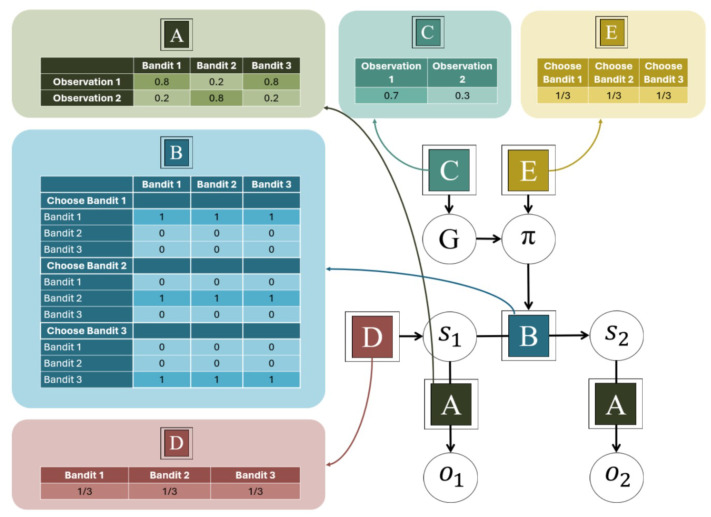
Specifications of the POMDP model.

A specific POMDP model suitable for MAB tasks was used to implement the individual active inference agents and also functioned as the generative model for the group-level agent. We here describe the details of this MAB-POMDP generative model (see Figure 2). Unless otherwise indicated, all parameter values and settings are the same for all internal agents, as well as for the group agent. The model has three environmental states *s*, one for each bandit arm. There are two types of observations *o*, 1 and 2, and three possible actions *u*, one for selecting each of the three bandit arms. Observation 1 is very likely for one of the arms and unlikely for the other two (as specified by the A matrix). Agents can choose which bandit arms to play, and their choice is deterministic, i.e., not subject to ‘motor errors’ (specified by a precise mapping in the B matrix). Agents have a preference for making observation 1 except when otherwise stated (specified by the C matrix) and have uniform priors over states and policies (D and E matrices). The policy posterior precision γ was 16, and the policy length is 2. With simplicity in mind, we do not include parameter learning but instead equip the agents with accurate beliefs about the outcome probabilities, thereby modelling behaviour at the point when learning has successfully converged. Since this means that there is nothing to learn about the environment, agents’ action selection is driven only by the pragmatic value in Equation (2).

### 2.2. Computational Cognitive Modelling

Computational cognitive modelling [64,65,66] is a methodology used within fields such as computational psychiatry [16], mathematical psychology [67], and model-based cognitive neuroscience [68] to formally describe and understand mental processes underlying behaviour. This is performed by constructing formal models of mental processes—such as belief updating and action selection—and how they generate behaviour on a given experimental task. These formal models can be used to simulate behaviour, dependent on some chosen parameter values. They can also be fitted to experimental data, a process which consists of using Bayesian inference, often approximated using Markov Chain Monte Carlo (MCMC) [64] or variational [69] methods, to estimate the most likely parameter values given some observed behaviour. Notably, the choice of model family and structure is made by the modeller on the theoretical grounds that the model is able to produce empirically observed behaviour (by successfully solving the experimental task). When multiple models are proposed, they can be compared by various approximations to their model evidence in what is called Bayesian model comparison or by other approaches such as prior and posterior predictive checks [66]. To test whether parameter inference is reliable, it is common to perform a parameter recovery check, which consists of simulating behaviour using a range of different known parameter values and then estimating those parameters based on the behaviour to see how well the estimates approximate the true parameter values. In this work, we fit a POMDP active inference model (Section 2.1) using the standard approaches described above—not to experimentally observed behaviour but to the behaviour of a simulated group agent. Note that, with brevity in mind, we do not perform model comparisons with other possible model structures, since the model we use is well suited for the task and standard in the active inference literature.

### 2.3. Cognitive Modelling for Collective Agents

**Figure 3:**
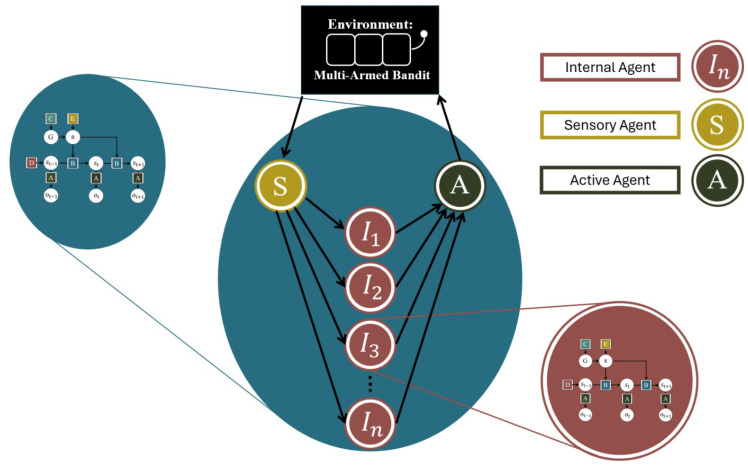
The group agent structure. The group agent consists of sensory agents S that pass on their observations to internal agents $I_{n}$. Internal agents’ actions are aggregated by an active agent A, which in turn affect the MAB environment. Internal agents are constructed as POMDP active inference agents, and a POMDP active inference model is fitted to the group agent’s behaviour.

In order to apply cognitive modelling to the group agent, the individual agents must be positioned in a Markov blanket structure. Here, we do not engage with *how* the agents come to be structured in a Markov blanket. Instead, for simplicity, we assume this process has already taken place and manually place the agents in an appropriate structure. Additionally, we do not introduce a separation of temporal scales between individual and group levels. The group agent (Figure 3) thus consists of (i) a set of *sensory agents* (in this case one) who observe the environment and acts accordingly; (ii) a set of *internal agents*, who observe the actions of the sensory agent and act accordingly; and (iii) a set of *active agents* (in this case one), who observes the actions of (some of) the internal and/or sensory agents (in this case all the internal agents) and whose actions affect the environment. The internal agents are all constructed as active inference agents. Owing to the simplicity of the setup, the POMDP-type generative model described in Section 2.1 is suitable for the individual agents as well as for the group agent performing the MAB task. This is because the internal agents receive and produce exactly the kind of observations and actions that a MAB task also would produce (similar to the predetermined Markov blanket structure and the lack of separation of time scales, this is not generally the case; see Section 4 for a discussion of ways in which to extend this setup). The sensory and active agents could easily be implemented as active inference agents equipped with appropriate generative models. However, for simplicity, we use a simple rule-based approximation to construct these agents. The sensory agent is thus a simple information transfer agent, i.e., it simply copies the input it gets from the environment and passes it on to the internal agents. The active agent is likewise kept as a simple aggregator, who observes the actions of all the internal agents and chooses an action with a probability proportional to the number of times it has been made by an internal agent (thereby functioning as a probabilistic voting mechanism).

The observations of the sensory agents and the actions of the active agents comprise the blanket states of the group agent. They correspond to its observations and actions, respectively, and are used to estimate the parameters of the generative model of the group agent, as described in Section 2.2. This makes it possible to compare the inferred parameter values of the group agents with those of the individual agents constituting it.

### 2.4. Simulation Experiments

We conducted a series of four simulation experiments that all share the same basic setup and simulation environment, each introducing a variation from the basic setup. For simplicity, we here focus on a single parameter at the group level which has a clear effect on behaviour: the action precision α. α is implemented as the temperature of a softmax over the agent’s action probabilities. Thus, agents with higher α will be more likely to select the action which is optimal relative to their preferences and current beliefs about the environment. Lower α will lead to more stochastic (or “noisy”) behaviour. As a preliminary, we performed a parameter recovery study to confirm that the α parameter can accurately be inferred. We simulated behaviour using α values between 0 and 2. In the recovery study and in the simulation experiments, we used a Multi-Armed Bandit task (MAB) with three bandits. The probabilities of (the generally preferred) outcome 1 for the three bandits were 0.8, 0.2, and 0.2. Because α is non-negative, we used a wide half-normal distribution as prior (mean = 0, SD = 4, truncated to be non-negative). When comparing generative values with estimates, we used the median of the parameter posteriors as a point estimate.

**Figure A1:**
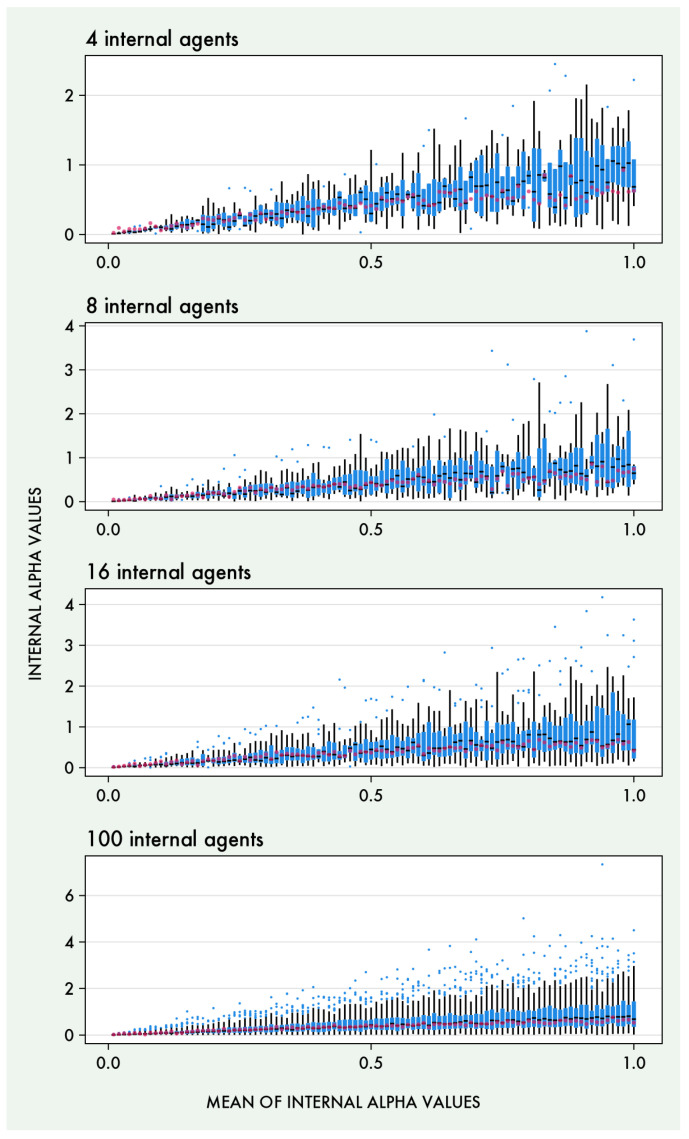
Distributions of α values across internal agents when using varying α values.

In the first simulation experiment, we made all internal agents identical and compared the α values of the internal agents to that of the group-agent. In the second experiment, we let α values vary between agents and compared the mean of the internal agents’ α values with the group α. We constructed the α values so as to control the mean of the distribution of internal agents’ parameter values. This was executed by drawing a set of weights from a Dirichlet distribution with sufficient statistics $\alpha_{i}=1.5,i=1,2\ldots n$, (where $\alpha_{i}$ designates the Dirichlet parameter, not to be confused with the parameter from the active inference model). These weights were multiplied by the number of internal agents and the desired mean in order to create the action precision α values for the active inference agents (see Figure A1 for the resulting distribution of α across internal agents). For the third experiment, we changed the active agent so that it no longer probabilistically aggregated votes but instead always chose the option with the most votes. For the fourth experiment, we let the prior preferences of the internal agents vary. We implemented this by drawing the relative preference for observation 1, for each internal agent, from a beta distribution with sufficient statistics α=0.8,β=0.8 (where α designates the Beta parameter, not to be confused with the parameter from the active inference model). For each experiment, we ran the simulation separately with 4, 8, 16, and 100 internal agents. We use α values for internal agents between 0 and 1. The goal of the third and fourth experiments was to investigate relations between the group α and other characteristics of the internal agents (other than their α value).

## 3. Results

### 3.1. Parameter Recovery

**Figure 4:**
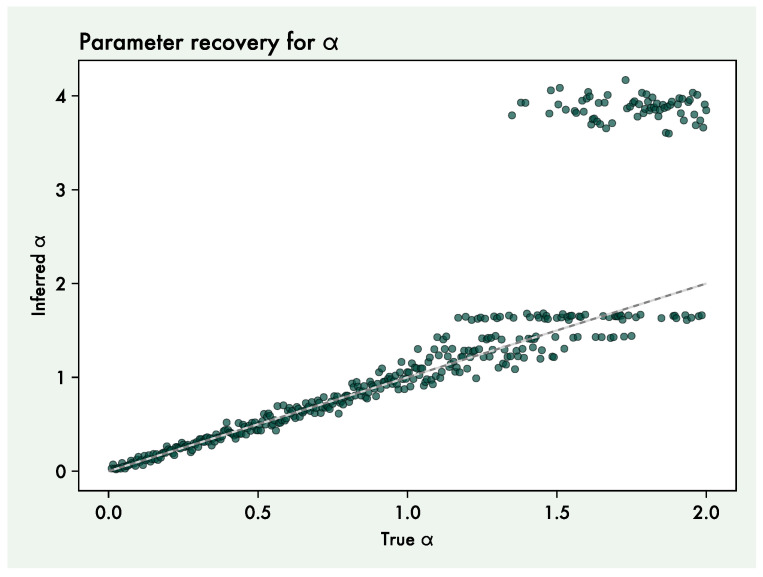
Parameter recovery results for the α (action precision) parameter. Each point is a recovery attempt, with the *x*-axis representing the generative α value of the agent and the *y*-axis representing the inferred value. The grey line depicts the identity function where recovery is perfect and is not a regression line.

The parameter recovery results shows that α can well be recovered in the range between 0 and 1 (Figure 4). The variance of inferred alphas increase at true α values above 1, with higher values resulting in inferred α values at around 2 or 4, irrespective of the true value. This indicates that α cannot be recovered well at values higher than 1. The efficiency of estimating parameters such as α depends on there being discernible consequences for behaviour when varying the parameter. When α is large, behaviour becomes largely deterministic so that there is no effect of further increasing α. In the following, we therefore do not use values above 1.

### 3.2. Simulation Experiments

**Figure 5:**
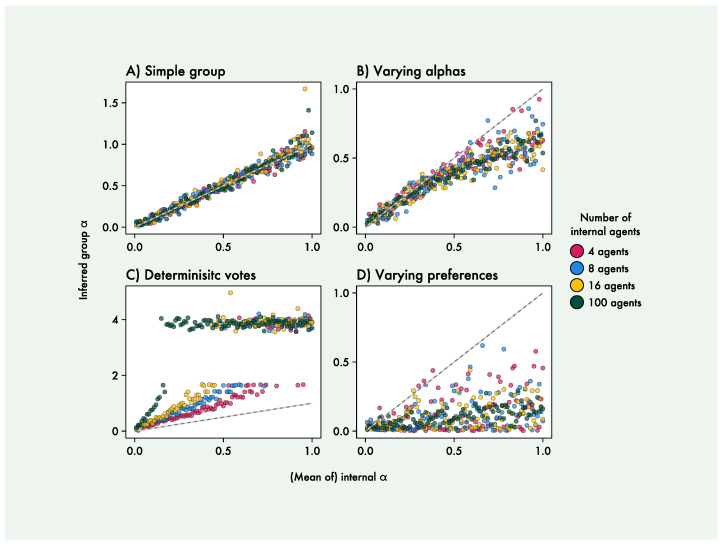
Results of the four simulation experiments. In (**A**), all individual agents have the same α values, which becomes identical to the group-level α. In (**B**), agents had varying α values constructed to have a specified mean, leading to a sub-linear relation between the group-level and average individual α. In (**C**), the active agent deterministically, as opposed to stochastically, selects the action with the most votes, leading to group-level agents with high action precision α. In (**D**), internal agents have conflicting preferences, leading to noisy low-α group-level agents. The inferred α value of the group agent is displayed against the mean of the internal agents’ α value. Each point is a single simulation, with colours distinguishing between different numbers of internal agents (red = 4 agents, blue = 8 agents, yellow = 16 agents, green = 100 agents). Grey lines depict the identity function, where the group α is identical to the mean of the internal agents’ α value, and is not a regression line.

The results of the four simulation experiments are shown in Figure 5. We see that the group α in the first experiment is equal to the shared α of the internal agents. In the second experiment, we find that the inferred group α scales with the mean of the internal agent α values, although sub-linearly, and that having larger numbers of internal agents results in less variance in the group α values. In the third experiment, we see that the deterministic voting aggregation leads to much steeper increases in group α as internal agent α increases, with the steepness further increasing as the number of internal agents increases. This quickly reaches α values high enough to display the distinct pattern of clustering at around 4, the part of parameter space where α no longer can be inferred well. For the fourth experiment, we find that internal agents with varying preferences lead to much lower group α values, and that higher numbers of internal agents again lead to less variance in the group α values.

## 4. Discussion

We have presented simulation methods and numerical studies of collective active inference with the generative models specified at the individual as well as ensemble levels and at corresponding spatiotemporal scales. This enabled us to investigate how the active inference process at the group level is influenced by the (inter-)active inference of individual agents. To make this possible, we employed methods from cognitive modelling, fitting an active inference model to the behaviour of the emergent group-level agent (i.e., blanket states).

As a proof-of-principle, we created a simple group of agents collaborating on a Multi-Armed Bandit (MAB) task and focused on the action precision parameter α of the group-level agent. We found that, with the POMDP generative model apt for Multi-Armed Bandit tasks, α recovered well in a range between 0 and 1. At higher α values, actions became fully deterministic to the point where different values had no influence on behaviour, making inference converge on values around 4 (the specific level is directly determined by the standard deviation of the prior). We therefore limited further analysis to α levels between 0 and 1 for internal agents. We then proceeded to simulate group behaviour and estimated α levels of group agents. We found that the group-level agent had the same α as the constituent agents when they all had the same value (Figure 5A). This was expected in this specific case where agents simply voted on the action that the group should take, aggregating identical agents results in a larger-scale version of that same agent.

When the α levels of individual agents vary, the group level agent enacts an α value similar to the mean of the individual agents (Figure 5B); essentially, the generative model of the larger level agent became an unweighted Bayesian model average of the constituent agents. The group-level α scaled sub-linearly with the average of the individual agents’ α values—this sub-linear relation is possibly related to the way the internal agent α values are constructed. Firstly, the internal agent α values must vary around the specified mean but are simultaneously bounded to be non-negative. In order to avoid extreme values, they are therefore sampled from a non-uniform Dirichlet distribution, which leads them to be non-symmetrically distributed with more α values below the mean than above it (Figure 5B). If the group-level α reflects the majority, rather than the mean, of the internal agents’ α values, this could lead to the observed sub-linear relation. In addition to this, differences in higher α values are less distinguishable behaviourally. This means that it is possible to increase the α values of some internal agents (and therefore the mean of the distribution) without increasing the inferred group-level α, also possibly contributing to a sub-linear scaling.

We also showcase two examples of how varying other aspects than α of the individual agents can lead to changes in the group-level α value. First, we see that a deterministic vote aggregation (where the action with the most votes is always chosen, as opposed to being chosen with a probability equal to the proportion of votes it received) led to markedly higher α values in the group-level agent (Figure 5C). The group level α now scaled super-linearly with the mean of the individual agents’ α values, with the slope increasing with the number of internal agents, now quickly leading to group α values reaching the upper bound of identifiability, where actions are fully deterministic. This was likely due to the diminishing effect of the internal agents’ stochasticity on the deterministic voting aggregation as the number of agents increases. This can be interpreted as a simple consequence of the law of large numbers, where the variance of a statistical estimator decreases as the number of samples increases, such that the ‘optimal’ action (highest-value) is chosen more reliably as the number of agents increases. Finally, we see that when individual agents had varying preferences, the group α was strongly reduced (Figure 5D). This was due to the agents’ votes now contradicting each other, resulting in a much more stochastic active agent and therefore a lower α for the group-level agent. We also see that larger amounts of internal agents reduced the variances in group α estimates. This was due to the way we had constructed the set of preferences, making it more likely to have a balanced set of contradicting preferences for higher numbers of internal agents.

In summary, this represents an example of how to investigate relations between individual and group-level emergent generative models of active inference agents. This makes it possible formally to relate all three levels—*within-agent*, *between-agent* and *group-as-agent* levels—simultaneously. In the last section, we discuss the limitations of our method, and ways in which it could be extended in the future.

### Applications and Extensions

We deliberately used a simple group agent which was constructed with a predefined Markov blanket structure, and an environment for which it is simple to formulate an appropriate generative model, which can also be used for internal agents. However, the method we used, in principle, applies to a much broader range of scenarios, namely, whenever there is an identifiable Markov blanket at the group level.

The method inherits a set of limitations from relying on methods from computational cognitive modelling. Most importantly, it infers the generative model of the group-agent from its observable behaviour (i.e., blanket states). This is only possible insofar as the behaviour is informative about the generative model; when several generative models (or parameter values) result in the same behaviour, it is not possible to distinguish between them. In our example, this is the case when α values are high enough that action becomes fully deterministic, so variations in α cannot be distinguished. There are other types of degeneracy: a uniform preference prior would lead to random behaviour indistinguishable from that associated with a very low α value, for example. Additionally, as in most computational modelling, there is an in principle infinite space of possible generative models that the emergent agent might have. Since searching this space is not practically possible, the approach relies on the modeller specifying a model structure, or a set of model structures to compare with Bayesian model selection. This, in turn, requires an environment for which a suitable generative model is identifiable. Notably, it is not actually an a priori necessary that the emergent group agent *has* a suitable generative model of the environment, especially in non-evolved settings or when a group agent fails to act appropriately; however, it is often in practice assumed that agents have environment-appropriate generative models. Finally, even if a generative model is identifiable, all the challenges of performing approximate Bayesian inference (with Markov-Chain Monte Carlo methods or with other approaches) apply here, such as the amount of computational resources required to fit models or the risk that samplers might not be able successfully to converge when fitting the model. Methods for addressing these types of problems apply here [64] except that simulation means that large quantities of data can be generated to make model fitting more precise, and that environments can be designed specifically to allow for distinguishing between different generative models. A challenge specific to this method is that a group-level Markov blanket has to be defined clearly, specifically requiring that sensory and active states can be defined for which it is possible to define a suitable generative model.

Despite these limitations, a cognitive modelling-based approach offers a tractable way to access the generative model of emergent agents with unknown generative models of the environment, making it possible to compare them to the generative models of individual constituent agents. This approach enables describing more than just the *spatially* self-similar nature of nested Markov blankets by elucidating the *cognitively* or *computationally* fractal nature of nested agents making inference over their environment. Notably, this approach is applicable to computational models of cognition in general, although it is particularly theoretically compatible with active inference modelling. Cognitive computational modelling in general assumes a Markov blanket structure (with the observable behaviour of an agent consisting of its observations and actions).

There are multiple ways to extend the simulations reported here, where internal agents within a static Markov blanket vote for a group action to be taken. One might add parameter learning, so as to allow inferring the consequences of one’s actions over time, such as is usually the goal in Multi-Armed Bandit tasks. One might also infer on other parameters than α for the group-agent, such as γ, the contents of the various matrices, learning rates for parameter learning, etc. One could also implement the sensory and active agents as active inference agents proper. This would facilitate going beyond simple aggregator and information transmission agents, for example, including sensory agents that can distort information about the environment or multiple (perhaps competing) agents in either role. It would also be possible to let agents take actions that reflect their level of certainty (which could lead to the group-agent becoming certainty-weighted Bayesian model average of the internal agents and would further facilitate exploration of the effect of individual-agent certainty dynamics on the group agent). It is also of interest to change the communication structure of the internal agents so that, instead of simple voting, agents interact in a more or less complex fashion, like a network structure where only a subset of internal agents pass their actions directly on to the active agents. Finally, one can also investigate beyond the simple POMDP-model used here, including more complex POMDP’s, continuous state space active inference models, or combinations thereof. The Multi-Armed Bandit task POMDP is general enough that it can be used for the single agents as well as for the constituent agent. However, this is not in any way required. Indeed, internal agents will often find themselves in very different environments from that of the group, mandating different generative models. This could also include models with explicit Theory of Mind, allowing agents explicitly to infer whether other agents are collaborating or competing, and whether active and sensory agents might for example have reasons to be untruthful. This would also allow agents to have explicit “altruistic” preference priors that include other agents’ reaching their goals. From here, it is not difficult to replace the Multi-Armed Bandit task environment with another group-agent, for example letting the two groups compete in game-theoretic tasks as with single agents. This lends itself easily to greater-than-two-scales simulations of group-agents constituted by sets of other group-agents, in turn made up of individual agents, etc.

There would also be value in applying this method to other types of simulations, including those with dynamically emerging Markov blankets or where dynamics at individual and group levels occur at different temporal scales. Many of the simulations reviewed in Section 1.2 do not have explicit environments for the group-agent, which makes it difficult to specify group-level generative models. It is not, however, difficult to imagine fitting a generative model to the behaviour of the active inferants [53] as if they were a single agents solving the T-maze task; the active inference fish school [54] as if it was a single fish-like agent; or the multiple Markov-blanketed cell groups that come together to create groups of groups of cells [22] as if each group was a single cell. In this way, one might investigate the relationship between *how* collective active inference agents emerge and the generative models they imply. One might in principle even apply the same methods to deep neural network structures like variational auto-encoders in order to infer implicit beliefs of the network.

There are also other approaches that could be of value to pursue in this context in addition to simply comparing parameter values (or generative model types) of individual and group-level agents. One obvious direction would be to look at the relations between dynamically changing beliefs (or prediction errors, etc.) at the group-level on one hand, and the equally dynamic beliefs or actions of the internal agents on the other—in the same way one might compare belief states to neural activity in model-based computational neuroscience [68]. Insofar as this were successful, it would help elucidate the relationship between internal (agent) state dynamics and emergent active inference processes, perhaps allowing for later reversing the inference to use internal state dynamics to help make inference about group-level beliefs even in the absence of observable environments. One might also combine the method with evolutionary algorithms, potentially at different scales; for example, one could test the hypothesis that selection pressure at the group-level fosters altruistic preferences in individual members, or attention to the needs of the group, while selection on the individual level fosters a stronger orientation (or stronger preference prior) toward accomplishing one’s own goals. One would also be able to distinguish the average success (or negative free energy) of the individual agents *as distinct from* the negative free energy of the group agent; this would be an interesting numerical study because the variational free energy is an extensive quantity. This means that the joint free energy of a group or ensemble is simply the sum of the individual free energies (i.e., if every member of the ensemble fulfils its prior preferences, then the group should also act as if it were fulfilling its prior preference). Whether this means the free energy under a group level generative model corresponds to the sum of free energies under individual generative models is an outstanding question.

One might also extend the detection of group-level Markov blankets; they could, for example, exist at slower timescales (as detectable for example by the renormalization group method [70]). There might also be ways to integrate multiple states as active or sensory states at the group level (perhaps in the fashion of integrated information, as in [71] or [72]). One might for example take the central mass of the active inference fish school [54] as the group-level active state, although it is not immediately obvious that this could be considered an active blanket state in the usual sense. Finally, one could also use this method for other types of systems with unknown generative models, such as animals or artificial systems, as well as laboratory-grown organoids and “dishbrains” [73]. In general, this method could be used to further the field of active inference and applications of the Free Energy Principle, as well as multi-scale adaptive processes, by providing access to the scale-free cognitive and computational processes they imply.

## Data Availability

The original data presented in the study are openly available in OSF at https://osf.io/6z3bd/.

## Abbreviations

The following abbreviations are used in this manuscript:

FEP	Free Energy Principle
POMDP	Partially Observed Markov Decision Process
MAB	Multi-Armed Bandit task
